# The Sulphuretted Hydrogen Gas Case

**Published:** 1851-07

**Authors:** 


### THE SULPHURETTED HYDROGEN GAS CASE.

In the article on this subject, in the present number, no allusion is made to the corroborative testimony of Prof. Lewis Rogers, for the new statements in relation to the treatment and results of the case. That gentleman was absent from the city when the article was written, but since his return he has read it, and says the statements concerning the case, during our connection with it, coincide with his recollections of it. He, like myself, was ignorant of Dr. Powell's advice in the case, until told of it by that gentleman. B.

